# Clinical relevance of non-tuberculous mycobacteria isolated from respiratory specimens: seven year experience in a UK hospital

**DOI:** 10.1038/s41598-018-37350-8

**Published:** 2019-02-11

**Authors:** H. F. Schiff, S. Jones, A. Achaiah, A. Pereira, G. Stait, B. Green

**Affiliations:** 1 Clinical and Experimental Sciences, University of Southampton, Southampton General Hospital, Tremona Road, Southampton, SO16 6YD UK; 2 Department of Respiratory Medicine, Queen Alexandra Hospital, Southwick Hill Road, Cosham, Portsmouth, PO6 3LY UK

## Abstract

The frequency of isolation of non-tuberculous mycobacteria (NTM) species from respiratory specimens is increasing, however the clinical relevance of such identifications vary by mycobacterial species and geographical location. A retrospective study of 853 NTM isolates from respiratory samples from 386 patients over seven years was performed. Clinical records and radiographic information were examined. Clinical significance was assessed by American Thoracic Society diagnostic criteria. 25% of all patients with respiratory isolates met criteria for non-tuberculous mycobacterial pulmonary disease (NTM-PD). Significant symptoms were weight loss, fever, night sweats, productive cough and haemoptysis. HIV co-infection was a significant risk factor for disease. Cavities, nodules and tree-in-bud were significant radiographic findings. *Mycobacterium avium* complex (MAC) were the dominant species isolated from this patient cohort. *Mycobacterium abscessus* (*M. abscessus*) was the species most likely to cause clinically significant disease and be sputum smear positive, thus warranting particular attention.

## Introduction

The frequency of nontuberculous mycobacteria (NTM) isolation from respiratory samples and NTM pulmonary disease (NTM-PD) is increasing in the UK^1,2^ and many other countries worldwide^3,4^. In part this is explainable by improved laboratory methods and increasing clinician awareness, however, it is widely accepted to represent a true increase in disease incidence^5^. Marked geographical variability in the prevalence of NTM-PD has also been shown, with additional geographical variation in the predominant strains causing disease^6^. NTM species also differ in their pathogenicity, with a higher propensity to cause disease in patients with impaired immunity. This can be either locally impaired immunity due to pre-existing lung disease or systemic, such as with haematological malignancy, immunosuppressive treatment or HIV/AIDS^7^. NTM are divided into slow growing and rapid growing based on their growth rate in culture. *Mycobacterium abscessus (M. abscessus)* is a rapid growing species, forming colonies on subculture in seven days or fewer. Whereas *Mycobacterium intracellulare (M. intracellulare)* and *Mycobacterium avium (M. avium)* subspecies, encompassed within the *Mycobacterium avium* complex (MAC), are slow-growing mycobacteria requiring more than seven days to form mature colonies on subculture^8^.

Following a smear positive sputum culture for acid fast bacilli, the presence of NTM requires differentiation from *Mycobacterium tuberculosis* (Mtb), particularly in the presence of cavitatory lung disease and in areas endemic for Mtb infection^9,10^. NTM are ubiquitous environmental organisms whereas Mtb cannot survive outside of a human host, with the resultant critical difference that the isolation of NTM from a respiratory specimen does not always represent disease. Whereas in contrast, a single isolate of Mtb by culture should be interpreted as representing pathogenic infection^11^.

To determine the clinical relevance of a positive NTM culture from a respiratory specimen, transient or persistent colonisation must be distinguished from infection, the latter indicating tissue invasion and the potential for consequent progressive NTM-PD. Criteria for the diagnosis of NTM-PD was defined in the American Thoracic Society (ATS) and Infectious Disease Society of America (IDSA) statement on the diagnosis, treatment and prevention of nontuberculous mycobacterial diseases in 2007^8^. Unlike Mtb infection where one positive culture is sufficient to indicate clinically significant infection, to meet diagnostic criteria for NTM-PD the patient must have characteristic symptoms, compatible radiology and two or more positive sputum samples of the same NTM species or one positive bronchial wash/lavage or compatible histopathological findings with one positive culture. Other potential causes of pulmonary disease must also be excluded. These diagnostic criteria are summarised in Table 1.

We performed a retrospective study of NTM isolates from respiratory specimens over a 7-year period from 2007 to 2014, to determine local epidemiology, clinical relevance of NTM isolates and to evaluate clinical practice. The medical records of patients from whom NTM were isolated were reviewed, including demographics, symptoms, predisposing conditions and radiological reports.

## Results

### Frequency of isolated NTM species

From 2007 to 2014 there were 853 NTM isolates from 386 patients in respiratory specimens at Queen Alexandra Hospital, Portsmouth, UK. 88% (748 of 853) NTM isolates were from sputum culture, 12% (104 of 853) were from broncho-alveolar lavage (BAL) and one sample was a lung swab. The most commonly isolated species were

**Table 1:** Summary of the ATS/IDSA diagnostic criteria for pulmonary non-tuberculous mycobacterial infection, adapted from^8^.

**Clinical**
• Pulmonary symptoms **AND**
• Appropriate exclusion of other diagnoses
**Microbiological**
• Positive culture results from at least two separate expectorated sputum samples **OR**
• Positive culture results from at least one bronchial wash or lavage **OR**
• Transbronchial or other lung biopsy with mycobacterial histopathological features and positive culture for NTM **OR** biopsy showing mycobacterial histopathological features and one or more sputum or bronchial washings that are culture positive for NTM
**Radiological**
• Nodular or cavitatory opacities on chest radiograph **OR**
• A thoracic CT scan that shows multifocal bronchiectasis with multiple small nodules

*M.intracellulare* and *M.avium*, accounting for 31% (267 of 853) and 21% (181 of 853) of isolates respectively. Further species isolated are shown by frequency in Table 2.

### Diagnosis of NTM–PD by ATS criteria

Of the 386 patients from whom 853 NTM isolates were found, 17 patients were excluded from analysis due to lack of clinical information, leaving 836 samples from 369 patients. In total there were samples from 444 clinical episodes, of which 25% (112) met the ATS 2007 diagnostic criteria and are referred to as ‘ATS positive’. 75% (332 of 444) clinical episodes did not meet ATS diagnostic criteria and were termed ‘ATS negative’.

**Table 2:** Mycobacterial species isolated from respiratory specimens by frequency.

Mycobacterial species	Isolates n (%)	Number smear positive	Number of clinical episodes	Percentage smear positive by clinical episode (%)
*Intracellulare*	267 (31.30)	8	124	33.33
*Avium*	181 (21.22)	5	75	6.67
*Gordonae*	130 (15.24)	1	109	0.92
*Abscessus*	72 (8.44)	12	19	63.16
*Chelonae*	72 (8.44)	8	45	17.78
*Xenopi*	67 (7.85)	4	36	11.11
*Malmoense*	40 (4.69)	3	18	16.67
*Kansasii*	16 (1.88)	1	11	9.09
*Mucogenicum*	8 (0.94)	0	7	0.00

Isolates are shown as n (%) from most frequent to least frequent isolates. The number of smear positive isolates and clinical episodes by mycobacterial species are shown. Smear positivity by clinical episode is expressed as a percentage.

### Clinical characteristics

Patient demographics, symptoms and predisposing conditions are summarised in Table 3. There were more females in the ATS positive group (59%) than males. The majority of NTM isolates in both groups were first isolates of that species and were isolated from sputum culture. Additionally significantly more isolates were obtained in the ATS positive group from BAL samples than in the ATS negative group. Statistically significant symptoms of the ATS positive group were weight loss, fever, night sweats, haemoptysis and productive cough. The majority of patients in both groups had underlying respiratory disease, however there was no significant association with a particular respiratory diagnosis in this cohort. Inhaled and oral steroid use were more common in the ATS positive group compared to the ATS negative group, however this did not reach statistical significance.

**Table 3:** Patient demographics, symptoms, predisposing conditions, underlying respiratory diagnoses and radiographic features by ATS diagnostic group.

Characteristics	ATS Positive (n = 112)	ATS Negative (n = 332)	Total (n = 444)
**Demography**			
Mean age (years)	65	67	
Female	59 (51.8)	150 (45.18)	209 (47.07)
**First isolate?**			
Yes	99 (86.8)	305 (91.87)	404 (90.99)
**Site**			
Sputum****	78 (61.90)	301 (90.12)	379 (82.39)
BAL****	48 (38.10)	33 (9.88)	81 (17.61)
**Symptoms**			
Productive cough*	89 (79.46)	227 (68.37)	316 (71.17)
Haemoptysis*	22 (19.64)	34 (10.24)	56 (12.61)
Dyspnoea	26 (23.21)	71 (21.39)	97 (21.85)
Fever**	14 (12.50)	12 (3.61)	26 (5.86)
Night sweats*	15 (13.39)	19 (5.72)	34 (7.66)
Weight loss****	40 (35.71)	53 (15.96)	93 (20.95)
Fatigue	6 (5.36)	16 (4.82)	22 (4.95)
**Underlying lung disease**			
Yes	99 (88.39)	281 (84.64)	380 (85.59)
TB	11 (9.82)	39 (11.75)	50 (11.26)
Bronchiectasis	43 (38.39)	146 (43.98)	189 (42.57)
Asthma	19 (16.96)	63 (18.98)	82 (18.47)
COPD	50 (44.64)	124 (37.35)	174 (39.19)
Pulmonary Fibrosis	6 (5.36)	16 (4.82)	22 (4.95)
Lung carcinoma	—	16 (4.82)	16 (3.60)
Other^#^	10 (8.93)	20 (6.02)	30 (6.76)
**Predisposing conditions**			
Inhaled corticosteroids	68 (60.71)	181 (54.52)	249 (56.08)
Alcohol dependence	3 (2.68)	7 (2.11)	10 (2.25)
Regular oral steroid use	16 (14.29)	36 (10.84)	52 (11.71)
HIV infection*	6 (5.36)	4 (1.2)	10 (2.25)
Haematological malignancy	3 (2.68)	8 (2.41)	11 (2.48)
Anti-TNF treatment	1 (0.89)	8 (2.41)	9 (2.03)
Other immunosuppressants^∆^	4 (3.57)	12 (3.61)	16 (3.60)
**Radiological features**			
No thoracic CT available	5 (4.46)	91 (27.41)	96 (21.62)
Tree-in-bud****	23 (20.52)	11 (3.31)	34 (7.66)
Nodules****	33 (29.46)	30 (9.04)	63 (14.19)
Cavities****	51 (45.54)	30 (9.04)	81 (18.24)
Emphysema	5 (4.46)	20 (6.02)	25 (5.63)
Bronchiectasis	22 (19.64)	73 (21.99)	21.40)

Results are shown as n (%).

*P < 0.05, **p < 0.005, ****p < 0.0001.

^#^Other respiratory conditions: aspergillosis lung disease (n = 17), Churg-Strauss syndrome/EGPA eosinophilic granulomatosis with polyangiitis (n = 4) tracheobronchitis (n = 1), chronic bronchiolitis (n = 1), obliterative bronchiolitis (n = 1) cystic fibrosis (n = 5).

^∆^Other immunosuppressants: chemotherapy (n = 7), methotrexate (n = 4), sulfasalazine (n = 2), mycophenylate (n = 1) azathioprine (n = 1), undefined (n = 1).

### Microbiological features

**Figure 1:**
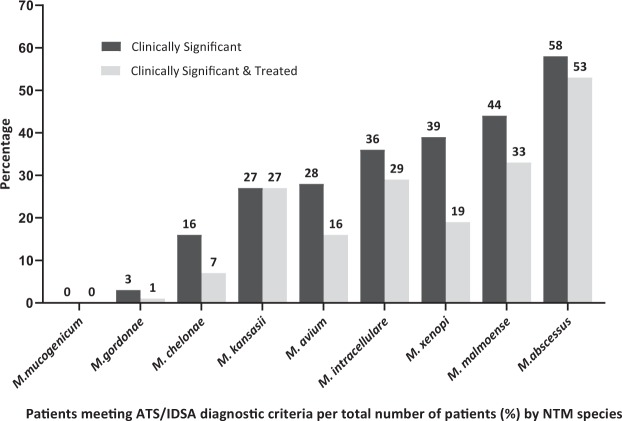
Clinical Relevance of NTM Isolates. Percentage of NTM isolates meeting ATS diagnostic criteria are shown by NTM species with comparative percentages of treated cases. Isolates are shown by increasing clinical relevance.

The most commonly isolated NTMs, *M. intracellulare* and *M. avium*, were clinically significant by ATS/IDSA criteria in 36% and 28% of clinical episodes, shown in Fig. 1. The most clinically significant NTM species isolated by ATS criteria was *M. abscessus*, meeting diagnostic criteria in 58% of cases. Additionally, *M. abscessus* was the most likely of all NTM species isolated to be sputum smear positive (63% of cases), as shown in Table 2.

### Radiological features

Thoracic computerised tomography (CT) scan reports, if present, were reviewed for both groups. Chest radiographs were not examined. 4% (5 of 112) of ATS positive patients did not have a CT scan available for analysis compared to 27% (91 of 332) in the ATS negative group. Cavities were the most frequent CT finding in the ATS positive group present in 46% (51 of 112) of cases. Nodules were present in 29% (33 of 112) and tree-in-bud were present in 21% (23 of 112) of ATS positive cases. All three features were strongly significantly associated with meeting ATS diagnostic criteria. 20% (22 of 112) of ATS positive cases had radiographic evidence of bronchiectasis and 4% (5 of 112) had emphysema.

### Treatment & Outcomes

91 clinical episodes were treated, of which 86% (78) met ATS diagnostic criteria. 69% (63 of 91) of clinical episodes were successfully treated. Treatment success was defined as symptomatic improvement and clearance of NTM from sputum culture. The mean treatment duration was 16.55 months, excluding that for *M. abscessus*. 16 patients died during the seven year period, 7 before starting treatment and the remainder during treatment. At the time of data collection 10 patients had relapsed following treatment, with the mean time to relapse of 10.1 months.

There were 34 ATS positive clinical episodes that were not treated. The most frequent reason cited for non-treatment was that symptoms were judged not severe enough to warrant treatment in 44% (15 of 34) cases. 20% of patients died before any treatment, 12% were treated for a separate pulmonary infection, 3% were judged too frail for treatment and in the remaining 21% the reasons for not treating were not documented or unclear.

There were 13 ATS negative clinical episodes that were treated despite not meeting diagnostic criteria. 5 did not meet radiographic criteria, 4 were treated on the basis of a single sputum sample and 4 were already on treatment for a different NTM species.

## Discussion

To our knowledge this is the first study to report the isolation frequency of NTM species from a coastal city in England. Our study demonstrated that the most frequently isolated NTM species were *M.intracellulare* and *M.avium*. This differs from observations in other non-coastal UK cities, for example previous retrospective studies in London showed that *Mycobacterium kansasii (M. kansasii) or Mycobacterium xenopi (M. xenopi)* were the dominant organisms^12,13^. NTM disease rates are known to vary widely depending on geographical location, and differences have also been observed between inland and coastal regions within other European countries, for example Croatia^14^.

25% of isolates by clinical episode had NTM-PD according to the ATS diagnostic criteria, which is similar to previously published studies in areas of the Netherlands, Canada & Korea^15–17^. Although the proportion of isolates meeting diagnostic criteria for NTM-PD more recently have varied widely from 5.5% in the entirety of Croatia to as high as 66% in a tertiary centre in China^18^.

The clinical significance of isolates differs widely between species, as illustrated in Fig. 1. *M. abscessus* was the most clinically relevant isolate reflecting NTM-PD in over 50% of cases. The ATS diagnostic criteria outline the recommended minimal diagnostic evaluation for NTM-PD for all species, however our data provide support for making the diagnostic criteria more stringent for species to the left of Fig. 1 such as *Mycobacterium mucogenicum* and *Mycobacterium gordonae* which are very infrequently clinically significant. Conversely for species that are clinically significant in more than 50% of cases, a diagnosis of NTM-PD may be warranted after a single positive culture. For the species of intermediate clinical significance (30–50% range), once diagnostic criteria have been reached, the decision to commence treatment should be made on an individual basis. However, NTM treatment regimens are lengthy, complex and can have attendant drug side effects. Therefore treating clinicians may pragmatically opt for a second culture confirmation of more clinically significant NTM isolates before embarking on a treatment course.

Our study has observed cases of ‘under treatment’ despite meeting diagnostic criteria. The most common reason for not commencing treatment was based on clinician judgement of disease severity, balanced against the implications of drug treatment. Cases of ‘over treatment’ were also observed which could be interpreted as unnecessary treatment and potential patient harm, however could also represent clinical circumstances not captured by our study.

We demonstrate highly statistically significant associations between meeting ATS criteria and weight loss in addition to fever, night sweats, haemoptysis and productive cough, although none of these symptoms are specific for a diagnosis of NTM-PD in isolation. Additionally the CT findings of cavities, nodules and tree-in-bud were highly significantly associated with NTM-PD according to the ATS diagnostic criteria. Although radiological criteria for NTM-PD can be met with a chest radiograph alone, the vast majority (>80%) of patients in both ATS positive and ATS negative groups had underlying lung disease. Cavities and nodules can be challenging to distinguish on a chest radiograph in the presence of underlying parenchymal lung disease. In addition the tree-in-bud phenomenon, representing endobronchial spread of infection in NTM-PD, is not visible on plain radiographs. The fact that 95% of patients meeting diagnostic criteria had a thoracic CT in our centre underlines the challenges of making a diagnosis of NTM-PD and reflects good specialist practice.

*M. abscessus* accounts for 80% of rapidly growing mycobacterial respiratory isolates^8^ and is inherently multidrug resistant so can be very challenging to treat. It was the most clinically significant mycobacterial isolate in our study which suggests that if it is isolated on one occasion only it is highly likely to represent pathogenic infection. Interestingly it was the most likely species by some margin to be sputum smear positive, highlighting the importance of microscopic sputum examination in addition to culture.

Despite a limited follow up period, a favourable treatment outcome in 69% is commensurate with published outcomes of treatment. In a review of practice in Canada and the USA treatment success rates ranged from 65–70% with a lower success rate perceived amongst non-expert practitioners compared to those with expertise in the management of NTM-PD^19^. Average treatment duration of 16.55 months is in line with 2007 ATS guidance and the more recently published 2017 British Thoracic Society guidelines for the management of NTM-PD^20^. These guidelines recommend that for *M. avium, M. intracellulare, M. xenopi, M. kansasii* and *M. malmoense* treatment is continued for one year following conversion to negative culture, which is expected in treatment responders at six months to one year following initiation of treatment. The recommended treatment for *M. abscessus* differs in that it includes an intravenous initiation phase, however the recommendation for treatment duration is the same as the previously mentioned species.

In conclusion, we have shown that the clinical relevance pattern of NTM isolates detected at our hospital laboratory differs from other areas of the UK and other countries worldwide. Whilst *M.intracellulare* and *M. avium* were the most commonly isolated species, *M. abscessus* was the most clinically relevant and therefore warrants particular attention. Adherence to the ATS diagnostic criteria was good and thoracic CT is a valuable diagnostic tool.

## Methods

This audit was registered and supported by the Portsmouth Hospitals NHS Trust Clinical Audit Department (Audit ID number 3558). The terms of agreement for the Portsmouth Hospitals NHS Trust Clinical Audit department were fully adhered to and only fully anonymised data is reported. Data was obtained as part of an audit of service and not part of a research study. Individual informed consent was not obtained.

All NTM strains isolated from respiratory specimens over a seven year period from January 2007 to December 2014 were retrospectively analysed. The medical records from whom NTM strains were isolated were retrospectively reviewed where available.

Microbiological samples were processed by Portsmouth Hospital National Health Service Trust Microbiology Laboratory in accordance with relevant guidelines and regulations. Sputum smear microscopy was performed using auramine staining and then examining for fluorescence under ultraviolet light. Liquid mycobacterial cultures were performed using the bioMérieux/BacT culture system. When a liquid culture flagged for possible growth, modified Ziehl-Neelsen staining was used to confirm mycobacterial growth. Once mycobacterial growth was confirmed, samples were sent to the National Mycobacterium Reference Laboratory South United Kingdom for full identification and sensitivities.

Patients from whom multiple samples were isolated were analysed by clinical episode, defined as isolation of a new species of NTM, a new positive sample after one year, development of new symptoms or new radiological change. If samples spanned more than one year with no change in clinical condition and the decision made not to treat, this was defined as one clinical episode. If multiple NTM species were isolated from samples of one individual, each new species was counted as a new clinical episode.

Statistical analyses were performed using Graphpad Prism v7.03. Fisher’s exact test was used for contingency table analyses of categorical variables. P values of less than 0.05 were considered to indicate statistical significance.
